# What factors have impact on glucocorticoid replacement in adrenal insufficiency: a real-life study

**DOI:** 10.1007/s40618-020-01386-3

**Published:** 2020-08-10

**Authors:** S. Puglisi, A. Rossini, I. Tabaro, S. Cannavò, F. Ferrau’, M. Ragonese, G. Borretta, M. Pellegrino, F. Dughera, A. Parisi, A. Latina, A. Pia, M. Terzolo, G. Reimondo

**Affiliations:** 1 Internal Medicine, Department of Clinical and Biological Sciences, University of Turin, Regione Gonzole 10, 10043 Orbassano, Italy; 2 Endocrinology and Diabetes Unit, ASST Papa Giovanni XXIII, Bergamo, Italy; 3 Department of Human Pathology ‘G.Barresi’, University of Messina, Messina, Italy; 4 Division of Endocrinology, AO S. Croce E Carle, Cuneo, Italy

**Keywords:** Hypoadrenalism, Addison, Hydrocortisone, Fludrocortisone, Management

## Abstract

**Purpose:**

The impact of patient’s characteristics on glucocorticoid (GC) replacement therapy in adrenal insufficiency (AI) is poorly evaluated. Aims of this study were to assess the influence of sex and body weight on GC dosing and to describe the choice of GC in AI of different etiologies.

**Methods:**

We retrospectively evaluated hydrocortisone (HC) equivalent total daily dose (HC-TDD) and per-kg-daily dose (HC-KDD) in 203 patients (104 primary AI [pAI], 99 secondary AI [sAI]) followed up for ≥ 12 months. They were treated with HC, modified-release HC (MRHC) or cortisone acetate (CA) and fludrocortisone acetate (FCA) in pAI.

**Results:**

At baseline, CA was preferred both in pAI and sAI; at last visit, MRHC was most used in pAI (49%) and CA in sAI (73.7%). Comparing the last visit with baseline, in pAI, HC-TDD and HC-KDD were significantly lower (*p* = 0.04 and *p* = 0.006, respectively), while FCA doses increased during follow-up (*p* = 0.02). The reduction of HC-TDD and HC-KDD was particularly relevant for pAI women (*p* = 0.04 and *p* = 0.002, respectively). In sAI patients, no change of HC-KDD and HC-TDD was observed, and we found a correlation between weight and HC-TDD in males (*r* 0.35, *p* = 0.02).

**Conclusions:**

Our real-life study demonstrated the influence of etiology of AI on the type of GC used, a weight-based tailoring in sAI, a likely overdosage of GC treatment in pAI women at the start of treatment and the possibility to successfully increase FCA avoiding GC over-treatment. These observations could inform the usual clinical practice.

## Introduction

Adrenal insufficiency (AI) is a rare but potentially life-threatening condition, characterized by impaired cortisol secretion due to a primary loss of function of the adrenal cortex [pAI] or secondary to a dysfunction of the hypothalamic–pituitary–adrenal axis [sAI]. The prevalence of pAI is lower than sAI (93–140 per million vs 150–280 per million, respectively) [1]. Both pAI and sAI patients need life-long treatment with glucocorticoids (GC) that, only in pAI, should be coupled with a mineralocorticoid analogue. Unfortunately, the long-term management of therapy is tricky, due to several issues.

Primarily, the physiological pattern of cortisol secretion is difficult to mirror using the conventional oral replacement therapy regimens [immediate-release hydrocortisone (HC), cortisone acetate (CA) or prednisolone] [2]. For this reason, a new formulation of once daily HC, defined as modified-release hydrocortisone (MRHC), has been recently introduced [3, 4]. MRHC use was associated with better quality of life and improved metabolic parameters compared with conventional GC regimens [5, 6]. However, a reliable biomarker to assess the adequacy of GC treatment in AI has not yet been identified and the dosage adjustments, whatever GC therapy is used, are guided only by the evaluation of clinical parameters and the personal judgment of the physician.

It is known that also patients on optimum replacement regimes are affected by more than a twofold increase in the standardized mortality ratio [7–10]. The reason of increased mortality is still unclear, but it is likely that underestimated conditions of under- and over-treatment could play a role. Indeed, chronic exposure to glucocorticoid over-replacement is associated with impaired glucose metabolism, central adiposity, higher cardiovascular risk and bone complications [11, 12], while an under-treatment causes adrenal crises, which can be potentially fatal [13, 14].

Although GC replacement is mainly based on clinical practice, the impact of patient's characteristics on daily GC requirement is poorly evaluated. There are few studies assessing the role of body weight on GC therapy. In 2004, Mah et al. reported that weight was the most important predictor of HC clearance and recommended weight-adjusted HC dosing [15]. More recently, Debono et al. stated that a weight‐related thrice‐daily dosing regimen is the best approach to tailoring hydrocortisone dose to meet patient needs and reproducing the cortisol circadian rhythm [16]. Few studies investigated sex-related differences in GC replacement, and reported conflicting results [17–19]. Moreover, there is a paucity of data regarding the choice of GC treatments for AI in real-world clinical practice.

Therefore, the primary aim of this study was to assess the influence of sex and body weight on dosing GC in patients with AI of different etiology and the secondary objective was to provide a survey of the use of different GC formulations (MRHC or other standard replacement therapy) in pAI and sAI.

## Patients and methods

### Patients

We retrospectively analyzed 203 consecutive patients with an established diagnosis of adrenal insufficiency, who were referred to three tertiary centers (San Luigi Gonzaga Hospital—University of Turin; Santa Croce e Carle Hospital of Cuneo; Gaetano Martino Hospital–University of Messina), between January 1995 and December 2018. Inclusion criteria were age at diagnosis ≥ 18 years; follow-up at the same center ≥ 12 months; stable glucocorticoid replacement for at least 6 months prior to the last visit. Patients treated with long-term exogenous glucocorticoids were excluded.

### Methods

Medical records were reviewed to obtain clinical information and to validate the diagnosis of AI, based on low basal serum cortisol (< 3 µg/dL) coupled with, in case of pAI, plasma levels of adrenocorticotropic hormone (ACTH) exceeding 100 pg/ml or abnormal response to the standard dose ACTH stimulation test (peak serum cortisol < 18 µg/dL) [20, 21].

The following data were collected from medical charts: date of birth, sex, etiology of AI, date of first visit (baseline), date of last visit and date of death. We retrieved, both at baseline and at last visit: age, weight, body mass index (BMI), waist, biochemical values (glycemia, glycated hemoglobin [HbA1c], total cholesterol, HDL cholesterol, triglycerides, electrolytes), cardiovascular or metabolic comorbidities (arterial hypertension, diabetes mellitus, dyslipidemia, cardiovascular events), and occurrence of adrenal crises. According to Hahner et al. [14], an adrenal crisis was defined as a worsening of the general condition with signs and symptoms of glucocorticoid and/or mineralocorticoid deficiency with at least two of the following conditions: (a) hypotension (systolic blood pressure < 100 mmHg), (b) nausea or vomiting, (c) severe fatigue, (d) documented hyponatremia, hyperkalemia, or hypoglycemia, and sub-sequent parenteral glucocorticoid administration. Adrenal crises that occurred before the diagnosis and start of replacement therapy were excluded from analysis. Presence of associated endocrine deficiencies [hypothyroidism, growth hormone (GH) deficiency, hypogonadism] and any replacement therapy were reported.

We registered the type of GC replacement therapy (CA, HC, MRHC; none of the patients were treated with prednisolone in our population), the frequency of administration and total daily dose both at baseline and at last visit. In pAI, total daily dose of mineralocorticoid (fludrocortisone acetate, FCA) replacement therapy was also reported. Cortisone acetate doses were calculated as HC-equivalent doses (mg cortisone acetate/1.25 = mg hydrocortisone) and we evaluated the HC total daily dose (HC-TDD) and per-kg-daily dose (HC-KDD).

The study was approved by the ethical committees of San Luigi Gonzaga Hospital—University of Turin, Santa Croce e Carle Hospital of Cuneo, Gaetano Martino Hospital—University of Messina, and it was conducted in accordance with the Declaration of Helsinki. The need to obtain informed consent from the study participants was waived due to the study's retrospective and observational nature.

### Statistical analysis

Categorical data are presented as counts and percentages. Continuous data are presented as means and standard deviations (SD). Differences in categorical variables were analyzed by means of the Chi-square test or Fisher test as appropriate, while differences in continuous variables by the Mann–Whitney *U* test. Correlation analyses were determined by calculating the Spearman’s *R* coefficient. All reported *P* values are two sided. *P* values less than 0.05 were considered as statistically significant. The statistical analyses were performed with *SPSS Statistics—version 25.0* IBM CORP**©**.

## Results

We analyzed 203 patients (118 females, F, 85 males, M) with a median age of 47 (range 18–88) years. Median follow-up was 75 (range 12–264) months. According to etiology of AI, we considered two different groups: 104 pAI patients (65 F, 39 M) and 99 sAI patients (53 F, 46 M).

At baseline, we did not observe any difference in clinical characteristics and comorbidities between pAI and sAI (Table 1), except for higher BMI and diastolic blood pressure levels in sAI and higher rate of adrenal crises in pAI. HC-TDD and HC-KDD were higher in pAI than in sAI.

**Table 1:** Baseline features of pAI and sAI

Characteristics	pAI (*n* = 104)	sAI (*n* = 99)	*p*
Gender *N* (%) Male Female	39 (37.5%) 65 (62.5%)	46 (46.5%) 53 (53.5%)	NS
Age at diagnosis (year) Mean ± SD	46.5 ± 15.4	50.0 ± 17.2	
Weight (kg) Mean ± SD	69.4 ± 16.5	74.1 ± 18.1	NS
BMI (kg/m^2^) Mean ± SD	25.3 ± 5.4	26.9 ± 5.7	**0.04**
Waist (cm) Mean ± SD	93.9 ± 17.1	96.8 ± 13.2	NS
Systolic blood pressure (mmHg) Mean ± SD	119 ± 17	123 ± 17	NS
Diastolic blood pressure (mmHg) Mean ± SD	75 ± 10	79 ± 11	**0.007**
Hypertension *N* (%)	21 (20.2%)	31 (31.3%)	NS
Diabetes mellitus *N* (%)	16 (15.4%)	11 (11.1%)	NS
Dyslipidemia *N* (%)	75 (72.1%)	69 (69.6%)	NS
Cardiovascular events *N* (%)	9 (8.6%)	7 (7.1%)	NS
Adrenal crisis *N* (%)	15 (14.4%)	3 (3.0%)	**0.009**
HC-TDD (mg/day) Mean ± SD	25.8 ± 7.4	21.1 ± 8.5	** < 0.001**
HC-KDD (mg/kg/day) Mean ± SD	0.44 ± 0.17	0.33 ± 0.14	** < 0.001**

Bold values indicate statistically significant values

*SD* standard deviation, *BMI* body mass index, *HC-TDD* hydrocortisone total daily dose, *HC-KDD* hydrocortisone per-kg-daily dose

**Fig. 1:**
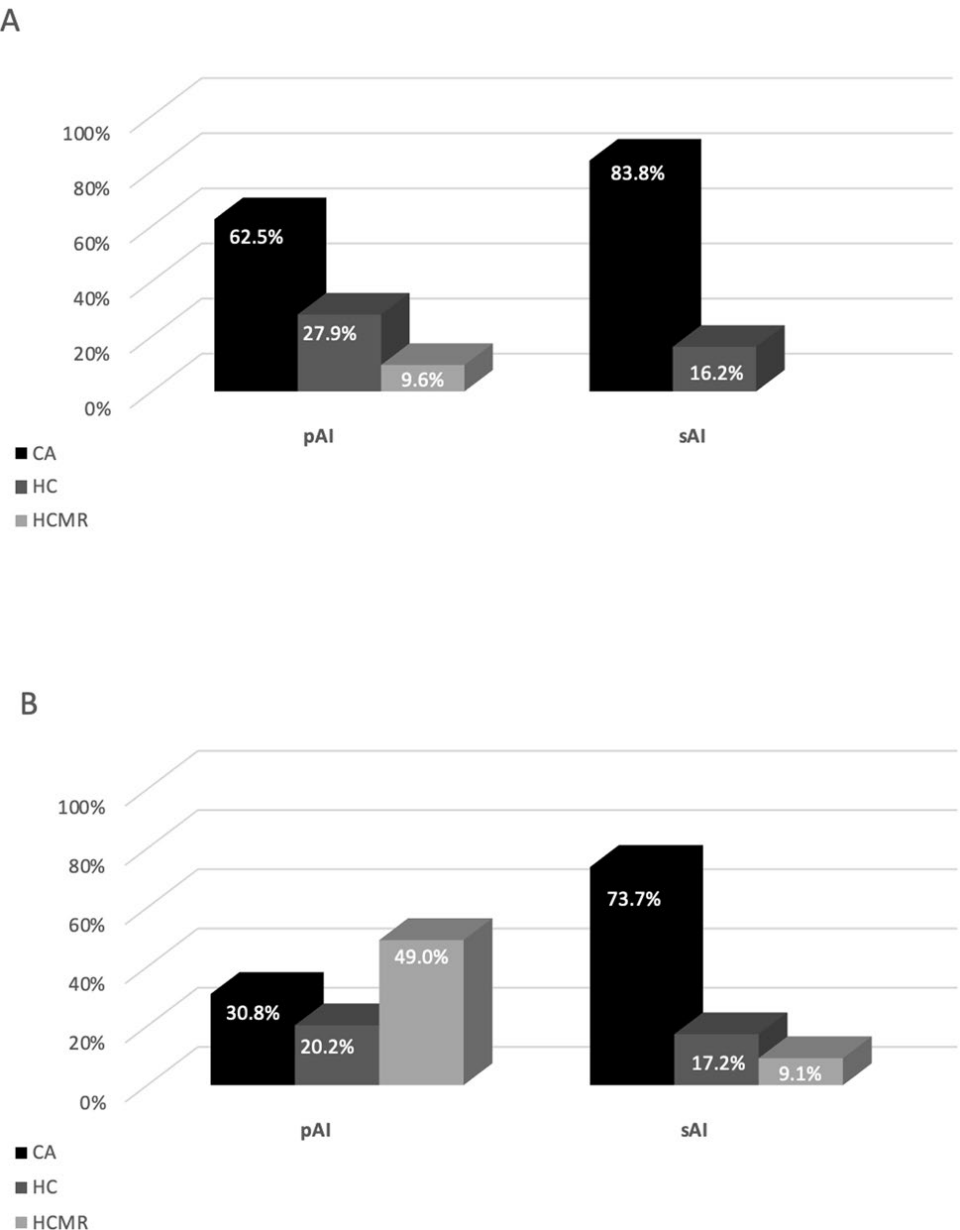
Comparison of glucocorticoid therapies between pAI and sAI groups, at baseline (**a**) at last visit (**b**)

At baseline, although either in pAI or in sAI CA was the preferred starting treatment, the use of HC was greater in pAI than in sAI (*p* < 0.001) (Fig. 1a). Ten patients with pAI started at baseline MRHC treatment. During follow-up, an additional subgroup of 53 patients (43 pAI and 10 sAI) has been switched to MRHC therapy. Three of them (2 pAI and 1 sAI) discontinued the new therapy after few months due to the limited clinical improvement and restarted the previous treatment with CA or HC. At last visit, the rate of patients in MRHC was higher in pAI than in sAI (*p* < 0.001) and CA was still preferred in sAI (Fig. 1b).

At last visit, we found higher weight, BMI, waist, systolic and diastolic blood pressure levels in sAI (Table 2), without difference in other comorbidities or occurrence of adrenal crises. HC-TDD and HC-KDD were still higher in pAI than in sAI.

**Table 2:** Characteristics of pAI and sAI at last visit

Characteristics	pAI (*n* = 104)	sAI (*n* = 99)	*p*
Gender *N* (%) Male Female	39 (37.5%) 65 (62.5%)	46 (46.5%) 53 (53.5%)	NS
Age at diagnosis (year) Mean ± SD	52.7 ± 15.6	56.1 ± 17.1	NS
Weight (kg) Mean ± SD	69.3 ± 14.9	74.7 ± 18.2	**0.02**
BMI (kg/m^2^) Mean ± SD	25.3 ± 5.0	27.2 ± 6.0	**0.02**
Waist (cm) Mean ± SD	91.0 ± 13.0	98.7 ± 11.0	** < 0.001**
Systolic blood pressure (mmHg) Mean ± SD	121 ± 15	126 ± 18	**0.03**
Diastolic blood pressure (mmHg) Mean ± SD	75 ± 10	78 ± 11	**0.04**
Hypertension *N* (%)	28 (26.9%)	39 (39.4%)	NS
Diabetes mellitus *N* (%)	15 (14.4%)	13 (13.1%)	NS
Dyslipidemia *N* (%)	57 (54.8%)	61 (61.6%)	NS
Cardiovascular events *N* (%)	5 (4.8%)	5 (5.1%)	NS
Adrenal crisis *N* (%)	11 (10.6%)	4 (4.0%)	NS
HC-TDD (mg/day) Mean ± SD	23.8 ± 6.7	20.7 ± 7.9	**0.003**
HC-KDD (mg/kg/day) Mean ± SD	0.38 ± 0.14	0.32 ± 0.13	**0.002**

Bold values indicate statistically significant values

*SD* standard deviation, *BMI* body mass index, *HC-TDD* hydrocortisone total daily dose, *HC-KDD* hydrocortisone per-kg-daily dose

Comparing the last visit with baseline, in pAI group weight and BMI were stable, but HC-TDD and HC-KDD were significantly lower (23.8 ± 6.7 *vs* 25.8 ± 7.4 mg/day, *p* = 0.04 and 0.38 ± 0.14 *vs* 0.44 ± 0.17 mg/kg/day, *p* = 0.006, respectively), while FCA doses increased during follow-up (0.06 ± 0.03 *vs* 0.07 ± 0.03 mg/kg/day, *p* = 0.02).

In sAI patients, weight, BMI, HC-KDD and HC-TDD were not different between baseline and last visit.

### Sex-related differences

At baseline, either in patients with pAI or sAI we did not observe any sex-related differences in clinical characteristics, biochemical value, comorbidities and occurrence of adrenal crises, except for higher weight in men, higher waist in men only in sAI and higher systolic blood pressure levels only in pAI men compared to pAI women (Tables 3, 4). While in sAI HC-KDD and HC-TDD were not significantly different between men and female (Table 4), in pAI HC-KDD was higher in F than in M (Table 3). Interestingly, only in M with sAI we have observed a correlation between weight and HC-TDD (*r* 0.57, *p* < 0.001). We have not observed any significant difference between M and F for the occurrence of hypogonadism, hypothyroidism or GH deficiency (Table 4).

**Table 3:** Baseline features of pAI, classified according to sex

Characteristics	F (*n* = 65)	M (*n* = 39)	*p*
Age at diagnosis (year) Mean ± SD	46.7 ± 14.9	46.4 ± 16.1	NS
Weight (kg) Mean ± SD	66.2 ± 17.5	74.8 ± 13.2	**0.01**
BMI (kg/m^2^) Mean ± SD	25.4 ± 6.2	25.1 ± 3.8	NS
Waist (cm) Mean ± SD	93.7 ± 19.4	94.0 ± 11.2	NS
Systolic blood pressure (mmHg) Mean ± SD	116 ± 18	125 ± 15	**0.01**
Diastolic blood pressure (mmHg) Mean ± SD	74.0 ± 10.7	77.0 ± 7.9	NS
Hypertension *N* (%)	12 (18.4%)	9 (23.1%)	NS
Diabetes mellitus *N* (%)	9 (13.8%)	7 (17.9%)	NS
Dyslipidemia *N* (%)	46 (70.7%)	29 (74.3%)	NS
Cardiovascular events *N* (%)	5 (7.7%)	4 (10.2%)	NS
Adrenal crisis *N* (%)	9 (13.8%)	6 (15.4%)	NS
HC-TDD (mg/day) Mean ± SD	25.8 ± 8.0	25.8 ± 6.2	NS
HC-KDD (mg/kg/day) Mean ± SD	0.47 ± 0.19	0.38 ± 0.17	**0.02**

Bold values indicate statistically significant values

*BMI* body mass index, *HC-TDD* hydrocortisone total daily dose, *HC-KDD* hydrocortisone per-kg-daily dose

**Table 4:** Baseline features of sAI, classified according to sex

Characteristics	*F* (*n* = 53)	*M* (*n* = 46)	*p*
Age at diagnosis (year) Mean ± SD	49.6 ± 15.7	50.1 ± 19.1	NS
Weight (kg) Mean ± SD	67.8 ± 16.3	81.3 ± 17.6	** < 0.001**
BMI (kg/m^2^) Mean ± SD	26.5 ± 6.3	27.6 ± 4.9	NS
Waist (cm) Mean ± SD	92.8 ± 14.8	100.2 ± 11.0	**0.007**
Systolic blood pressure (mmHg) Mean ± SD	121.8 ± 19.3	124.7 ± 19.3	NS
Diastolic blood pressure (mmHg) Mean ± SD	78.9 ± 10.8	78.5 ± 11.6	NS
Hypertension *N* (%)	15 (28.3%)	16 (34.8%)	NS
Diabetes mellitus *N* (%)	5 (9.4%)	6 (13.0%)	NS
Dyslipidemia *N* (%)	38 (71.7%)	31 (67.4%)	NS
Cardiovascular events *N* (%)	3 (5.6%)	4 (8.7%)	NS
Adrenal crisis *N* (%)	1 (1.9%)	2 (4.6%)	NS
Hypogonadism *N* (%)	39 (73.6%)	36 (78.3%)	NS
Hypothyroidism *N* (%)	42 (79.2%)	39 (84.8%)	NS
GH deficiency *N* (%)	26 (49.1%)	24 (52.2%)	NS
HC-TDD (mg/day)			
Mean ± SD	19.8 ± 8.3	22.6 ± 8.7	NS
HC-KDD (mg/kg/day)			
Mean ± SD	0.35 ± 0.16	0.31 ± 0.11	NS

Bold values indicate statistically significant values

*BMI* body mass index, *HC-TDD* hydrocortisone total daily dose, *HC-KDD* hydrocortisone per-kg-daily dose

At baseline, doses of FCA were similar between M and F and in both groups, weight and BMI were not correlated with FCA dose.

Comparing the last visit with baseline, in F with pAI weight was stable, but HC-TDD was significantly reduced (25.8 ± 8.0 *vs* 23.2 ± 6.3 mg/day, *p* = 0.04), as well as HC-KDD (0.47 ± 0.19 *vs* 0.38 ± 0.14 mg/kg/day, *p* = 0.002). Doses of FCA were similar to baseline.

In M with pAI, BMI, HC-KDD and HC-TDD were not significantly different between baseline and last visit.

At last visit, HC-KDD, HC-TDD and FCA doses were not significantly different between M and F and did not correlate with weight or BMI. Moreover, during the follow-up period, the occurrence of adrenal crises and comorbidities were comparable between groups and not significantly different comparing with baseline.

In patients with sAI at last visit, the weight of female was stable when compared to baseline, but HC-TDD was significantly lower in F than in M (F, 18.4 ± 6.9 *vs* M, 23.4 ± 8.1 mg/day, *p* = 0.001), without any change in HC-KDD. In male patients with sAI, BMI, HC-KDD and HC-TDD were not different between baseline and last visit. Also at last visit in sAI, we have not observed any significant difference between M and F for other pituitary deficiencies and all the patients were on adequate replacement treatment. Moreover, data were comparable with baseline.

At last visit in sAI, although we have not observed any significant differences of HC-TDD and HC-KDD between males and females, only in M we have demonstrated a correlation between weight and HC-TDD (*r* 0.35, *p* = 0.02).

Interestingly, only in male patients, BMI and waist circumference were higher in SAI than in PAI either at baseline (waist 100.2 ± 11.0 vs 94.0 ± 11.2 cm, *p* = 0.01, BMI 27.6 ± 4.9 vs 25.1 ± 3.8 kg/m^2^, *p* = 0.01) or at the last follow-up (waist 102.2 ± 10.7 vs 92.7 ± 10.5 cm, *p* = 0.001, BMI 27.3 ± 5.2 vs 24.9 ± 3.4 kg/m^2^, *p* = 0.001), while these parameters were comparable for female patients.

At last visit, comorbidities and occurrence of adrenal crises were comparable between groups and not significantly different comparing with baseline.

## Discussion

The replacement therapy of AI in clinical practice is characterized by a significant heterogeneity in type and dosage of the prescribed GCs [22]. To our knowledge, few works so far investigated the impact of patients’ characteristics on this heterogeneity. In our study, we evaluated how gender, weight, and AI etiology influenced the management of AI in three referral centers in Italy.

In our AI patients, CA was the preferred therapy. This is in contrast with previous published data reporting that in most countries HC is the drug of choice for adrenal insufficiency [17, 18, 23]. CA is biologically inactive, requiring conversion to cortisol by 11 bHSD1, and this may lead to a slight delay in achieving the peak of serum cortisol levels [24]. For this reason, HC is often preferred, although at present there are no data showing its superiority over CA [25]. On the other hand, it has been reported that CA is widely prescribed in Italy given that HC is not readily available and needs specific prescription by an endocrinologist [26, 27]. Therefore, the higher proportion of patient being treated with CA in our cohort reflects common clinical practice of our country, due to the prompt availability of CA.

In our study population, CA was more frequently prescribed to patients with secondary (sAI) than primary (pAI) adrenal insufficiency. To our knowledge, this is an unreported finding since most studies show that the etiology of AI does not influence the choice of treatment [18, 23]. Secondary AI is usually associated with other pituitary defects known to interfere with the conversion of CA to cortisol, particularly growth hormone (GH) deficiency [28]; thus, CA seems unsuitable in this setting. Despite that, some data suggest that CA may be a better option than HC in patients with sAI. Swords et al. [29] reported a lower 11 bHSD activity when hypopituitary patients were taking CA compared to those taking HC, thus leading to a more physiological tissue exposure to glucocorticoid. Moreover, Filipsson et al. [30] observed that sAI patients receiving HC displayed a higher risk of developing an adverse metabolic profile than those using CA. The prompt availability and these data should support the prevalent use of CA.

MRHC is a novel HC formulation designed to more closely mimic the circadian rhythm of cortisol secretion [3]. Several studies reported advantages of MRHC over the conventional GC formulations with regard to metabolic outcomes, QoL and compliance [4, 31, 32]. In our cohort, MRHC was rarely considered as starting treatment, but its use significantly increased over time and one third of patients were taking MRHC at the end of the study. The shift to MRHC was generally well tolerated except for three patients that preferred the previous treatment. The observation that patients with pAI were more frequently treated with MRHC than those with sAI both at baseline and at the end of the study is not completely unexpected since MRHC has been extensively studied only in pAI [33] while data in sAI are less conclusive. Mongioì et al. have recently reported a less beneficial effect on metabolic profile in sAI patients treated with MRHC [34], although also in these patients was reported a maintained improvement in the clinical condition and subjective well-being. Moreover, commercially available MRHC preparations are not always easy to tailor for the majority of sAI patients that require low–intermediate doses and costs are much higher with that formulation [27].

At baseline, patients with pAI were treated with higher doses of glucocorticoid than those with sAI, and this was confirmed at the end of the study despite a significant reduction of HC dose took place in pAI. This finding is in agreement with previous data showing that patients with sAI need lower replacement doses since they could maintain a residual cortisol secretion [35]. Patients with pAI are also more prone to receive supraphysiological doses of HC, and this is likely explained by the fear of adrenal crises. We cannot draw any conclusion in this regard since our study was not aimed at evaluating comorbidities. In our cohort, patients with sAI displayed higher BMI and blood pressure values than those with pAI, but sAI is more often associated to comorbidities that may impact the metabolic profile of the patients.

Weight is an important variable determining glucocorticoid clearance, and the use of weight-based regimens instead of fixed doses of HC has been advocated for the treatment of AI [15, 16]. In our patients, the replacement doses of GC correlated with weight in male patients with sAI but not in those with pAI. This could be due to a different fat distribution between the two groups observed at last visit and also at baseline, with a prevalence of central obesity in sAI male patients without any difference for female patients. It has been demonstrated that in obese subjects cortisol clearance show a correlation with intra-abdominal fat [36]; therefore, it is possible that in sAI patients, who are more frequently affected by overweight and obesity (due to concomitant pituitary hormones deficiency), weight could likely influence the dose of GC. Our data seem to demonstrate that this effect is more evident in males than in females. In sAI, glucocorticoid replacement is usually associated with therapies interfering with cortisol metabolism [27], and this was confirmed in our cohort since most patients with sAI were treated for other hormonal deficiencies. However, we have not observed a possible influence either of the hormonal deficiencies’ prevalence or the replacement treatment on the effect of different glucocorticoid replacement therapy on males and females.

So far, few studies evaluated the impact of gender on the replacement therapy of hypocortisolism. Iqbal et al. analyzed a population of 2648 AI patients from UK, showing that females were more often treated with long-acting glucocorticoid than males, but they did not report data about replacement doses [18]. Dalin et al. did not find differences between sexes in the body surface-adjusted HC dose in a population of more than 600 Swedish patients with autoimmune Addison disease [17], while Skov et al., in a similar cohort, reported a slightly higher total HC dose in males [19].

In our patients with pAI, females received at baseline a glucocorticoid dose adjusted for body weight significantly higher than males. This difference was no longer present at the end of the observational period, due to a significant reduction of the replacement doses in the female subgroup. Males and females with sAI were treated with a similar weight-adjusted glucocorticoid dose both at baseline and at the end of the study. Gender does not affect the clearance of hydrocortisone [15] and females display slightly lower serum cortisol levels compared to males [37]. Furthermore, Skov et al. recently reported that women treated with high glucocorticoid replacement doses were more prone to develop cardiovascular events than men [19]. Thus, the fact that in our study females with pAI were treated with higher GC doses compared to males seems mainly related to the use of standard replacement regimens in both sexes. Since males have a significantly higher weight than females, the use of comparable total glucocorticoid doses at baseline resulted in a possible over-replacement in females. By now, it became clear that the normal daily cortisol secretion is lower than once estimated [38] and several data emerged regarding the detrimental consequences of glucocorticoid over-replacement [30, 39], leading to a reduction in glucocorticoid doses for treatment of hypocortisolism [20, 21]. The significant reduction of glucocorticoid dose observed in our subgroup of females with pAI at the end of the study should be also in part explained by the introduction of the fixed doses of the MRHC treatment that induces to reconsider the total daily doses usually administered.

The majority of patients with pAI require mineralocorticoid replacement with FCA, even if some are well controlled with HC alone, with variables percentages in the different series [17, 19, 26, 40]. In our cohort, all patients with pAI were treated with FCA according to guidelines [20]. Few studies so far have described the characteristics of mineralocorticoid replacement in patients with AI [41]. Interestingly, Dalin et al. [17] reported a similar HC dose in patients with pAI irrespective of whether they were treated with FCA or not. Our data seem to diverge from this observation since in our population of patients with pAI the decrease in HC dose was paralleled by a significant increase in FCA dose. HC possess mineralocorticoid activity and HC over-treatment may offset mineralocorticoid replacement [42]. It is thus likely that the relatively high HC dose given to our pAI patients at baseline limited the need for FCA. The reduction of HC dose during follow-up seems to require a parallel increase of FCA to obtain adequate compensation.

The limitation of our study relates to its retrospective nature that may underestimate morbidity. However, the reduction of the administered HC doses does not seem to be accompanied by an increased frequency of adrenal crises or a worsen health profile. Instead, long-term beneficial effects may be expected on bone and CV events, reducing the number of patients in over-treatment. Moreover, although the GC replacement total daily doses were calculated as HC-equivalent doses, we cannot exclude a possible partial influence of the type of therapy, due to the different timing and pharmacokinetics.

In conclusion, our study provides an insight of the management of AI in the real clinical practice of three referral centers in Italy. We have shown that the diagnosis of primary or secondary AI influences replacement therapy of hypocortisolism either for the preferred drug or its dosage. A greater proportion of patients with sAI were treated with CA, while MRHC was mainly prescribed in patients with pAI. In patients with pAI, weight was not considered at the start of treatment, producing a potential over-replacement in females that was mitigated by the optimization of the therapy during follow-up. Since females with AI may be more exposed to the detrimental effects of glucocorticoids excess, our observation underlines the need of an accurate tailoring of treatment in these patients and the introduction of the MRHC should ease this aim. Moreover, in females pAI patients at diagnosis, we should consider lower starting doses than that usually prescribed in males to avoid potential long-term side effects. Moreover, our data suggest that in patients with pAI, variations in GC replacement should be associated with concomitant adjustments in FCA dosage. This observation may also lead to a review of the usual clinical practice of acting predominantly on HC dosages, while it may be indicated in many patients to also adjust FCA dosages to obtain optimal electrolyte compensation, avoiding HC over-treatment. On the other hand, patients with sAI were more frequently treated with weight-based glucocorticoid regimens. Our data could support the physician in the decisional process on replacement therapy in different settings increasing the accurate tailoring of treatment in each patient.
